# Ecological supports and reading experiences in rural XiZang: associations with primary students' social-emotional competencies

**DOI:** 10.3389/fpsyg.2026.1790046

**Published:** 2026-03-16

**Authors:** Shuangjiao Deng

**Affiliations:** Faculty of Education, Northeast Normal University, Changchun, Jilin, China

**Keywords:** cross-sectional study, ecological supports, reading experience, rural XiZang, social-emotional competencies, teacher-led reading

## Abstract

**Introduction:**

This study examined the associations between ecological support factors and social-emotional competencies (SEC) among 463 rural XiZang primary school students (Grades 4–6).

**Methods:**

Using a cross-sectional survey design, regression analyses, cluster analysis, and bootstrap-based mediation analyses were conducted to explore how ecological supports, reading experiences, and perceived instructional practices related to students' SEC.

**Results:**

Teacher Support showed the strongest and most consistent associations with SEC dimensions (*B* = 0.15–0.34, *p* < 0.05), while grade-stratified analyses revealed that sixth graders exhibited a distinct pattern in which Class Management and Family Support, rather than Teacher Support, were significantly associated with SEC. Students who reported prior experience with emotion-themed picture books showed significantly higher SEC scores across multiple dimensions. Cluster analysis identified three student profiles differing in levels of ecological support and SEC, with significant associations observed between profile membership and family economic status, parental marital status, and reading experience. Cross-sectional mediation analyses indicated that students' perceptions of teacher-led reading activities (Character Emotion Analysis and Emotion Regulation Instruction) showed significant indirect associations with the Teacher Support–SEC relationship, whereas perceptions of formal curricular courses (Moral and Legal Education, Psychological Health Education) did not.

**Discussion:**

These findings suggest that teacher-led reading practices may represent a salient correlate of social-emotional development in resource-constrained educational settings, though future longitudinal and experimental research is needed to establish causal relationships and evaluate intervention effectiveness.

## Introduction

Social-emotional competencies have become a central construct in educational psychology, reflecting a broad consensus that students' academic success depends not only on cognitive skills but also on their capacity to regulate emotions, manage social relationships, and engage productively in learning environments (Pekrun and Linnenbrink-Garcia, 2014; Zeidner et al., 2012). A substantial body of research published in leading journals has demonstrated that competencies such as emotional regulation, self-awareness, and interpersonal skills are robustly associated with academic achievement, classroom engagement, and school adjustment (Mega et al., 2014; Trentacosta and Izard, 2007; Cipriano et al., 2023). Among the various social contexts influencing these competencies, the teacher-student relationship has received particular attention. Supportive and responsive teacher interactions have been shown to promote students' emotional wellbeing while simultaneously enhancing academic performance (Roorda et al., 2011; Wubbels et al., 2015). As educational systems across the world increasingly emphasize holistic approaches to student development, identifying the ecological conditions that support social-emotional competencies has become a key agenda for contemporary educational psychology research (Zins and Elias, 2007; Oberle et al., 2020; Mahoney et al., 2021).

Despite considerable progress in understanding social-emotional learning in well-resourced educational environments, much less is known about how these competencies develop in rural, culturally distinct, and resource-constrained contexts (Yang et al., 2018). This gap is especially pronounced in research on minority and Indigenous student populations, where cultural norms, linguistic practices, and community structures may shape social-emotional development in ways that are insufficiently captured by dominant theoretical frameworks (Durlak et al., 2011; Lee and Zuilkowski, 2022). Rural XiZang represents a particularly underexamined context in this regard. Schools in this region typically operate in bilingual instructional settings, incorporate local cultural traditions that emphasize communal harmony and respect for elders, and face substantial structural challenges, including high teacher-student ratios and limited instructional resources (Postiglione et al., 2005). These distinctive ecological conditions raise important questions about whether established findings regarding teacher support, school climate, and family involvement can be generalized to such settings, or whether alternative patterns of association may emerge. Despite the theoretical and practical importance of these issues, systematic empirical research on the social-emotional competencies of students in rural XiZang remains extremely limited.

From an ecological perspective, children's social-emotional development is shaped by multiple, interrelated contextual systems that operate across different levels of the environment (Bronfenbrenner, 1979; Rosa and Tudge, 2013; Neal and Neal, 2013). Within school settings, several ecological supports have been consistently linked to students' social-emotional outcomes. Teacher-student relationships constitute one of the most extensively studied factors, with meta-analytic evidence indicating that teacher warmth, instructional support, and emotional responsiveness are positively associated with students' social-emotional adjustment and academic engagement (Roorda et al., 2011; Quin, 2017). School climate, encompassing institutional norms, perceptions of safety, and students' sense of belonging, has likewise been associated with higher levels of prosocial behavior, stronger emotional regulation, and fewer behavioral problems (Wang and Degol, 2016; Bear et al., 2015). Classroom management practices, including clear expectations and consistent routines, provide essential structural conditions that support the development of social-emotional skills (Korpershoek et al., 2016). Beyond the school context, family support, reflected in parental involvement, emotional availability, and socioeconomic resources, represents another critical ecological layer shaping children's social-emotional trajectories (Bradley and Corwyn, 2002). However, how these multiple ecological supports jointly relate to social-emotional competencies in culturally distinctive and resource-limited contexts such as rural XiZang remains largely unexplored. In addition, whether the relative importance of these factors differs across developmental stages, such as between younger and older primary school students, constitutes a further gap in the existing literature.

In addition to broader ecological supports, classroom-level pedagogical practices may also play a meaningful role in students' social-emotional development. Reading activities that incorporate emotional content have received increasing attention as a potentially accessible means of fostering empathy, perspective-taking, and emotional understanding (Mar et al., 2011; Wimmer et al., 2024). Through narrative engagement, students are able to explore characters' emotional experiences and moral dilemmas, which may enhance their own emotional awareness and interpersonal sensitivity (Kidd and Castano, 2013). In educational contexts where formal social-emotional learning curricula are limited in scope or difficult to implement with fidelity, teacher-guided reading activities may offer a practical alternative for embedding social-emotional content within everyday instruction (Lee, 2023). Nevertheless, empirical evidence on the association between students' reading experiences, particularly exposure to emotion-themed materials, and their social-emotional competencies in low-resource educational settings remains scarce. Moreover, little is known about whether students' perceptions of different instructional approaches, such as teacher-led reading activities compared with formal courses in moral or psychological education, are differentially associated with their social-emotional development.

Addressing these questions in the rural XiZang context also requires measurement instruments that are both psychometrically robust and culturally appropriate. Although established measures such as the Social Emotional Assets and Resilience Scale (Nese et al., 2012) and the Social and Emotional Competence Questionnaire (Zhou and Ee, 2012) have been validated in other populations, their applicability to students in rural XiZang has not yet been empirically established. These students navigate bilingual educational environments, culturally specific value systems, and distinctive socioeconomic conditions that may influence how social-emotional competencies are expressed and reported. Establishing the reliability and factorial validity of adapted measurement tools is therefore a necessary methodological prerequisite for any substantive investigation of social-emotional development in this population.

Against this backdrop, the present study examines the ecological factors associated with social-emotional competencies among primary school students in rural XiZang, with particular attention to potential variation across grade levels and student background characteristics. Four research questions guide the study. First, do adapted measures of social-emotional competencies and ecological support factors demonstrate adequate reliability and factorial validity in a rural XiZang sample? Establishing measurement adequacy provides the methodological foundation for addressing the subsequent questions. Second, how are five ecological support factors, namely teacher support, school support, family support, classroom climate, and classroom management, associated with different dimensions of students' social-emotional competencies, and do these associations differ across grade levels? Third, how are students' reading experiences, specifically their exposure to emotion-themed picture books, related to their social-emotional competencies? Fourth, are students' perceptions of different instructional approaches, including teacher-led reading activities and formal curricular courses, differentially associated with their overall social-emotional competencies, and do these perceived practices partially account for the association between teacher support and social-emotional outcomes?

## Methods

This study employed a cross-sectional survey design to examine the associations between ecological support factors, reading experiences, and social-emotional competencies among primary school students in rural XiZang (Creswell and Creswell, 2018; Bethlehem, 2009).

### Participants

A total of 463 primary school students from Grades 4 to 6 were recruited from rural schools in Lhasa, XiZang. The sample comprised 31 percent fourth grade students, 40 percent fifth grade students, and 29 percent sixth grade students. Gender distribution was approximately balanced, with 48 percent male and 52 percent female participants. With respect to family structure, 16 percent of the students were only children, whereas 84 percent reported having one or more siblings. The distribution across grade levels, gender, and family structure yielded a heterogeneous sample suitable for examining social-emotional competencies and their ecological correlates in the rural XiZang context.

Sample size adequacy was evaluated based on the analytical requirements of the planned statistical procedures. For multiple regression analyses involving five predictors, power analyses conducted using G^*^Power indicated that a minimum sample size of approximately 92 participants was required to detect a medium effect size with an *f*-squared value of 0.15, an alpha level of 0.05, and statistical power of 0.80 (Green, 1991; Faul et al., 2009). For factor analytic procedures, methodological guidelines recommend a minimum subject-to-item ratio of 5 to 1 (Costello and Osborne, 2005; DeVellis and Thorpe, 2021; Westland, 2010). Given that the survey instrument contained 73 items, a minimum of 365 participants was required. The final sample of 463 students exceeded both criteria, providing sufficient statistical power for all planned analyses.

### Measures

Data were collected using the Elementary School Students social-emotional competencies Survey, a self-report questionnaire consisting of four sections. The overall instrument demonstrated excellent internal consistency, with a Cronbach alpha coefficient of 0.95. Descriptive statistics, including means, standard deviations, skewness, and kurtosis, were calculated for all scales. The factorial structure of the primary measures was examined using exploratory and confirmatory factor analyses, with detailed results reported in the Results section.

#### Section 1: demographic and background information

This section collected basic demographic and background information, including students' grade level, gender, only child status, and perceived family economic status. Students were also asked to indicate whether they had prior experience reading emotion-themed picture books, using a binary yes or no response format. This item served as a self-reported indicator of students' reading exposure and was not based on experimental manipulation or researcher-administered intervention.

#### Section 2: social-emotional competencies scale

The social-emotional competencies scale was developed through the adaptation and integration of items from established instruments, including the Social Emotional Assets and Resilience Scale (Nese et al., 2012), the Social and Emotional Competence Questionnaire (Zhou and Ee, 2012), and the Emotional and Social Competence Inventory (Boyatzis et al., 2000). Scale development was also informed by Goleman's theoretical framework of emotional intelligence (Goleman, 1996). All items were reviewed to ensure linguistic clarity and cultural appropriateness for students in rural XiZang and were translated into both Mandarin Chinese and Tibetan to accommodate the bilingual instructional context.

The final scale consisted of 29 items assessing six dimensions of social-emotional competencies. Emotional Regulation referred to students' ability to recognize, manage, and modulate their emotional states. Prosocial Values and Interpersonal Skills captured cooperative behaviors, empathy, and altruistic tendencies. Recognition of Others' Emotions assessed students' capacity to accurately interpret emotional cues from peers, teachers, and family members. Emotional Understanding reflected awareness of the causes and consequences of emotions across different situations. Self-Awareness and Expression indicated students' ability to identify and appropriately communicate their own emotions. Socially Normative Expression and Collaboration represented adherence to social norms and effective cooperation in group contexts. All items were rated on a five-point Likert scale ranging from one, strongly disagree, to five, strongly agree.

#### Section 3: ecological support factors scale

The Ecological Support Factors scale was developed based on existing instruments, including the Delaware School Climate Survey (Bear et al., 2014) and the Developmental Assets Profile (Scales, 2011), with adaptations made to reflect the local educational context. The scale comprised 44 items assessing five dimensions of ecological support. School Support captured institutional resources, policies, and leadership practices that contribute to students' sense of safety and belonging. Teacher Support reflected students' perceptions of teachers' warmth, instructional guidance, and emotional responsiveness. Classroom Climate assessed peer relationships, communication norms, and mutual respect within the classroom. Class Management evaluated students' perceptions of instructional organization, behavioral expectations, and classroom routines. Family Support captured perceived emotional, material, and motivational support from parents or guardians. All items were rated on a five-point Likert scale.

#### Section 4: curricular integration and implementation

This section included four items assessing students' perceptions of the extent to which different instructional approaches supported their emotional learning. Students evaluated their perceived learning experiences associated with two formal curricular courses, Moral and Legal Education and Psychological Health Education, as well as two forms of teacher-guided reading activities. One item assessed how frequently teachers guided students to analyze the emotional states of fictional characters during reading activities, referred to as Character Emotion Analysis. A second item assessed how frequently teachers helped students learn strategies for managing emotions through reading materials, referred to as Emotion Regulation Instruction. All items were rated on a five-point scale reflecting perceived frequency and perceived usefulness.

These items captured students' retrospective perceptions of instructional practices encountered in their regular classroom environments. They did not represent standardized interventions implemented by the research team, nor were students randomly assigned to different instructional conditions. Consequently, the data from this section reflect students' subjective evaluations of naturally occurring pedagogical practices rather than experimentally controlled instructional treatments.

### Procedure

Prior to data collection, a comprehensive review of relevant literature was conducted, along with preliminary field consultations, to identify key constructs and contextual factors related to social-emotional competencies among primary school students in the XiZang context. Based on established instruments described above, an initial version of the questionnaire was developed and subsequently reviewed to ensure cultural relevance and linguistic clarity. To accommodate the bilingual instructional environment of the participating schools, the questionnaire was prepared in both Mandarin Chinese and Tibetan.

Following consultation with and approval from school administrators and homeroom teachers, the questionnaire was administered electronically to students in Grades 4 to 6. Data collection was conducted during regular school hours under the supervision of classroom teachers. At the beginning of the survey, students were provided with age-appropriate explanations regarding the purpose of the study, the intended use of the data, and assurances of confidentiality. Students were informed that participation was voluntary and that they could withdraw at any time without penalty.

Given the minimal risk nature of the study and the age of the participants, passive parental consent was obtained through information letters distributed by the schools, and active assent was obtained from the students prior to participation. All study procedures were reviewed and approved by the Psychological Ethics Committee under Protocol No. PEC-2024-014.

A total of 480 questionnaires were collected. To ensure data quality, 17 responses were excluded due to excessive missing data, defined as more than 10 percent of items unanswered, or implausibly short completion times, defined as less than 5 min for a survey designed to require approximately 15–20 min to complete. These patterns suggested inattentive or careless responding. The final analytic sample consisted of 463 valid responses, resulting in a data retention rate of 96.46 percent.

### Data analysis strategy

A multi-stage analytical strategy was employed to address the four research questions (Tabachnick et al., 2013). All analyses were conducted using Python version 3.9 (Python Software Foundation, Wilmington, DE, United States) for exploratory factor analysis, confirmatory factor analysis, and cluster analysis, and R version 4.2 (R Foundation for Statistical Computing, Vienna, Austria) for multiple regression and mediation analyses. The threshold for statistical significance was set at *p* less than 0.05 for all inferential tests.

### Measurement validation

To address the first research question concerning measurement adequacy, exploratory factor analyses were conducted separately for the social-emotional competencies scale and the Ecological Support Factors scale to examine their underlying factor structures. Principal axis factoring with varimax rotation was applied. Items with weak primary factor loadings below 0.40 or substantial cross-loadings were considered for removal to improve construct clarity and measurement quality. Confirmatory factor analyses were then conducted to evaluate the fit of the hypothesized factor structures. Model fit was assessed using multiple indices, including the comparative fit index, with values of 0.90 or higher indicating acceptable fit, the root mean square error of approximation, with values of 0.08 or lower indicating acceptable fit, and chi-square statistics. Internal consistency reliability was assessed using Cronbach alpha coefficients for each subscale.

### Group comparisons

To provide preliminary descriptive insights into variation in social-emotional competencies across student subgroups, independent samples *t*-tests were conducted to compare social-emotional competencies scores by gender, only child status, and reading experience, defined as whether students reported having read emotion-themed picture books. One-way analyses of variance were conducted to examine differences across grade levels and categories of family economic status.

### Regression analyses

To address the second research question, multiple regression analyses were conducted to examine the associations between five ecological support factors, namely School Support, Teacher Support, Classroom Climate, Class Management, and Family Support, and each dimension of social-emotional competencies. Each social-emotional competencies dimension was entered as the dependent variable in separate regression models, with all five ecological support factors entered simultaneously as predictors. To explore potential developmental differences, additional regression analyses were conducted separately for students in Grades 4, 5, and 6, using total social-emotional competencies scores as the dependent variable.

### Cluster analysis

To identify distinct subgroups of students based on their profiles of social-emotional competencies and ecological supports, hierarchical cluster analysis was conducted using Ward's method with squared Euclidean distance measures. The optimal number of clusters was determined based on inspection of the dendrogram and the conceptual interpretability of the resulting cluster profiles. Chi-square tests of independence were subsequently conducted to examine associations between cluster membership and categorical background variables, including family economic status, parental marital status, and reading experience.

### Mediation analysis

To address the fourth research question, mediation analyses were conducted to examine whether students' perceptions of instructional practices were statistically associated with the relationship between Teacher Support and overall social-emotional competencies. Four parallel mediation models were tested, with Moral and Legal Education, Psychological Health Education, Character Emotion Analysis, and Emotion Regulation Instruction specified as potential mediators of the association between Teacher Support and social-emotional competencies. Mediation analyses were performed using a bootstrap procedure with 5,000 resamples to generate bias-corrected 95 percent confidence intervals for indirect effects (Hayes, 2018). An indirect effect was considered statistically significant if the corresponding confidence interval did not include zero.

It should be noted that these mediation analyses were based on cross-sectional data, which precludes causal inference. The assumed temporal ordering in the models cannot be empirically verified within the present design, and the results should therefore be interpreted as exploratory and hypothesis-generating.

## Results

### Measurement validation

Exploratory factor analysis (EFA) was conducted using principal axis factoring with varimax rotation to examine the dimensional structure of the Social-Emotional Competencies (SEC) scale and the Ecological Support Factors (ESF) scale. For the 29-item SEC scale, sampling adequacy was excellent (KMO = 0.949; Bartlett's test of sphericity: χ^2^ = 6992.71, *p* < 0.001). The analysis yielded a six-factor solution consistent with the hypothesized dimensions described in the Measures section. Most items exhibited primary factor loadings at or above 0.50, with minimal cross-loadings.

For the 44-item ESF scale, sampling adequacy was similarly excellent (KMO = 0.968; Bartlett's test: χ^2^ = 20949.47, *p* < 0.001). A clear five-factor structure emerged corresponding to the five hypothesized dimensions. Primary factor loadings were generally high, although a small number of items showed modest cross-loadings at this exploratory stage. Overall, the EFA results for both instruments were consistent with the theoretical frameworks guiding scale development.

Confirmatory factor analysis (CFA) was subsequently conducted to evaluate the hypothesized factor structures. For the six-factor SEC model, χ^2^(362) = 1235.26. Standardized factor loadings ranged from 0.63 to 0.73, with all items loading above 0.50 on their intended factors, providing support for the six-dimensional structure. For the five-factor ESF model, χ^2^(892) = 4095.03. Most standardized loadings exceeded 0.60, and no further item removal was warranted. These factor-analytic results established adequate measurement models for both scales, providing a psychometric foundation for subsequent analyses.

### Group differences in social-emotional competencies

Independent-samples *t*-tests and one-way ANOVAs were conducted to examine variation in SEC across demographic and background variables. No significant differences were found by gender or only-child status for any SEC dimension (all *p*s > 0.05).

**Figure 1:**
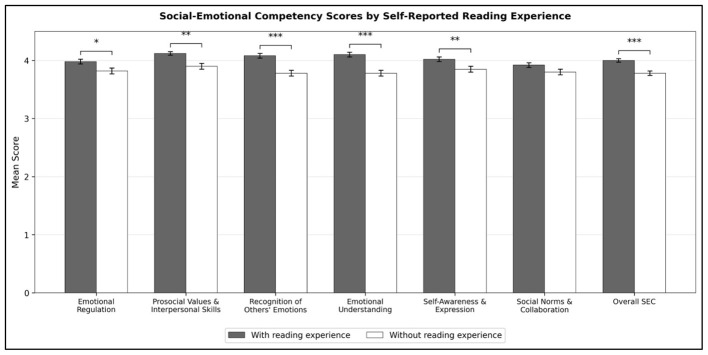
Social-emotional competency scores by self-reported reading experience. Error bars represent standard errors. *p < 0.05, **p < 0.01, ***p < 0.001.

However, significant differences were observed based on students' self-reported reading experience. Students who reported having previously read emotion-themed picture books scored significantly higher than those without such experience across multiple SEC dimensions (see Figure 1): Emotional Regulation (*t* = 2.43, *p* = 0.015), Prosocial Values and Interpersonal Skills (*t* = 3.14, *p* = 0.002), Recognition of Others' Emotions (*t* = 4.38, *p* < 0.001), Emotional Understanding (*t* = 3.80, *p* < 0.001), Self-Awareness and Expression (*t* = 2.91, *p* = 0.004), and overall SEC (*t* = 3.68, *p* < 0.001). The difference in Socially Normative Expression and Collaboration approached but did not reach statistical significance (*p* = 0.057). These findings indicate a consistent pattern of higher SEC scores among students reporting prior reading experience.

One-way ANOVA revealed modest grade-level differences. Although most SEC dimensions did not differ significantly across grades, sixth graders scored higher than fourth graders on Emotional Understanding [*F*_(2, 460)_ = 3.07, *p* = 0.048]. Family economic status was associated with broader differences: significant or marginally significant variation emerged for Prosocial Values and Interpersonal Skills (*p* = 0.028), Emotional Understanding (*p* = 0.035), and overall SEC (*p* = 0.050). Students from higher-income families tended to show higher scores on these dimensions, though economic status was not significantly associated with the remaining SEC dimensions.

### Associations between ecological support factors and social-emotional competencies

Six separate multiple regression analyses were conducted to examine how the five ESF dimensions were associated with each SEC dimension (see Table 1). Teacher Support showed the most consistent pattern of associations, reaching statistical significance for all six SEC dimensions: Emotional Regulation (*B* = 0.287, *p* < 0.001), Prosocial Values and Interpersonal Skills (*B* = 0.250, *p* < 0.001), Recognition of Others' Emotions (*B* = 0.216, *p* = 0.001), Emotional Understanding (*B* = 0.150, *p* = 0.018), Self-Awareness and Expression (*B* = 0.239, *p* < 0.001), and Socially Normative Expression and Collaboration (*B* = 0.338, *p* < 0.001). School Support was significantly associated with five of six dimensions (e.g., *B* = 0.118, *p* = 0.049 for Emotional Regulation; *B* = 0.197, *p* = 0.006 for Self-Awareness and Expression). Class Management showed significant associations with Prosocial Values and Interpersonal Skills (*B* = 0.144, *p* = 0.031), Recognition of Others' Emotions (*B* = 0.256, *p* = 0.005), and Emotional Understanding (*B* = 0.191, *p* = 0.026). Family Support was significantly associated with Emotional Regulation (*B* = 0.139, *p* = 0.001) and Socially Normative Expression and Collaboration (*B* = 0.096, *p* = 0.036). Classroom Climate did not show significant associations with any SEC dimension. Overall, Teacher Support and School Support demonstrated the most consistent positive associations with SEC dimensions.

**Table 1:** Multiple regressions predicting SCE dimensions.

**Predictor**	**SCE dimension**	***B***	**Std. error**	***t***	***p***
School support	Emotional regulation	0.118	0.060	1.973	0.049
Teacher support	Emotional regulation	0.287	0.055	5.191	< 0.001
Classroom climate	Emotional regulation	0.049	0.077	0.635	0.526
Class management	Emotional regulation	0.060	0.075	0.801	0.424
Family support	Emotional regulation	0.139	0.040	3.433	0.001
School support	Prosocial values	0.170	0.053	3.216	0.001
Teacher support	Prosocial values	0.250	0.049	5.097	< 0.001
Classroom climate	Prosocial values	0.084	0.068	1.235	0.218
Class management	Prosocial values	0.144	0.066	2.160	0.031
Family support	Prosocial values	0.011	0.036	0.310	0.757
School support	Others' emotion recognition	0.077	0.071	1.076	0.282
Teacher support	Others' emotion recognition	0.216	0.066	3.275	< 0.001
Classroom climate	Others' emotion recognition	−0.088	0.092	−0.960	0.338
Class management	Others' emotion recognition	0.256	0.090	2.854	0.005
Family support	Others' emotion recognition	0.009	0.048	0.194	0.846
School support	Emotional understanding	0.155	0.068	2.268	0.024
Teacher support	Emotional understanding	0.150	0.063	2.366	0.018
Classroom climate	Emotional understanding	0.063	0.088	0.719	0.473
Class management	Emotional understanding	0.191	0.086	2.227	0.026
Family support	Emotional understanding	−0.046	0.046	−0.990	0.323
School support	Self-awareness and expression	0.197	0.071	2.773	0.006
Teacher support	Self-awareness and expression	0.239	0.066	3.617	< 0.001
Classroom climate	Self-awareness and expression	0.017	0.091	0.183	0.855
Class management	Self-awareness and expression	0.008	0.090	0.094	0.925
Family support	Self-awareness and expression	0.036	0.048	0.750	0.454
School support	Social norms and collaboration	0.144	0.068	2.121	0.034
Teacher support	Social norms and collaboration	0.338	0.063	5.373	< 0.001
Classroom climate	Social norms and collaboration	−0.008	0.087	−0.096	0.923
Class management	Social norms and collaboration	0.107	0.085	1.257	0.209
Family support	Social norms and collaboration	0.096	0.046	2.104	0.036

### Grade-stratified regression analyses

To examine potential developmental differences, additional regression analyses were conducted separately for each grade level, with overall SEC as the dependent variable (see Table 2). Each model explained approximately 40%−45% of the variance in SEC scores.

**Table 2:** Regression of SCE_total by grade.

**Grade**	**Predictor**	***B***	**Std. error**	***t*-value**	***p***
G4	School support	0.154	0.082	1.878	0.063
G4	Teacher support	0.305	0.067	4.521	< 0.001
G4	Classroom climate	0.044	0.097	0.456	0.649
G4	Class management	−0.073	0.097	−0.753	0.453
G4	Family support	0.148	0.082	1.795	0.075
G5	School support	0.133	0.076	1.737	0.084
G5	Teacher support	0.226	0.081	2.809	0.006
G5	Classroom climate	0.034	0.108	0.315	0.753
G5	Class management	0.190	0.122	1.560	0.121
G5	Family support	0.000	0.046	0.001	0.999
G6	School support	0.131	0.117	1.118	0.266
G6	Teacher support	0.044	0.119	0.367	0.714
G6	Classroom climate	−0.157	0.172	−0.915	0.362
G6	Class management	0.453	0.155	2.928	0.004
G6	Family support	0.161	0.073	2.192	0.030

Among fourth graders [*F*_(5, 137)_ = 19.53, *p* < 0.001, adjusted *R*^2^ = 0.395], Teacher Support was the only factor significantly associated with SEC (*B* = 0.305, *p* < 0.001), while School Support (*B* = 0.154, *p* = 0.063) and Family Support (*B* = 0.148, *p* = 0.075) approached but did not reach significance. Neither Classroom Climate (*B* = 0.044, *p* = 0.649) nor Class Management (*B* = −0.073, *p* = 0.453) showed significant associations.

A similar pattern was observed for fifth graders [*F*_(5, 178)_ = 27.40, *p* < 0.001, adjusted *R*^2^ = 0.419], where Teacher Support remained significantly associated with SEC (*B* = 0.226, *p* = 0.006). School Support showed a marginally significant association (*B* = 0.133, *p* = 0.084), while other factors did not reach significance (*p*s ≥ 0.120).

The results for sixth graders, however, revealed a notably different pattern [*F*_(5, 130)_ = 21.57, *p* < 0.001, adjusted *R*^2^ = 0.433]. Class Management (*B* = 0.453, *p* = 0.004) and Family Support (*B* = 0.161, *p* = 0.030) were significantly associated with SEC, whereas Teacher Support (*B* = 0.044, *p* = 0.714) and School Support (*B* = 0.131, *p* = 0.266) were not. These findings suggest that the relative importance of different ecological factors may vary across developmental stages, with older students showing stronger associations between SEC and factors related to classroom structure and family environment.

### Cluster analysis

Cluster analysis was conducted to identify subgroups of students based on their combined SEC and ESF profiles. Three distinct clusters emerged. Cluster 1, labeled “High Support, High Competency,” comprised students with high scores on both SEC dimensions and ecological support factors. Cluster 2, “Moderate Support, Moderate Competency,” included students with mid-range scores on both sets of variables. Cluster 3, “Low Support, Low Competency,” consisted of students with relatively low scores on both SEC and ESF measures.

Chi-square tests of independence revealed significant associations between cluster membership and several background variables. Cluster membership was significantly associated with family economic status (χ^2^-test, *p* = 0.038), self-reported reading experience (*p* < 0.001), and parental marital status (*p* = 0.017). *Post-hoc* comparisons indicated that higher economic status primarily differentiated Cluster 1 from Cluster 2 (*p* = 0.015), while reading experience showed pronounced differences between Cluster 1 and both Clusters 2 and 3 (*p* < 0.001). Parental marital status differences were most evident between Cluster 1 and Cluster 3 (*p* = 0.021). No significant associations were found between cluster membership and gender (*p* = 0.479), grade level (*p* = 0.158), or only-child status (*p* = 0.743).

### Mediation analyses

**Figure 2:**
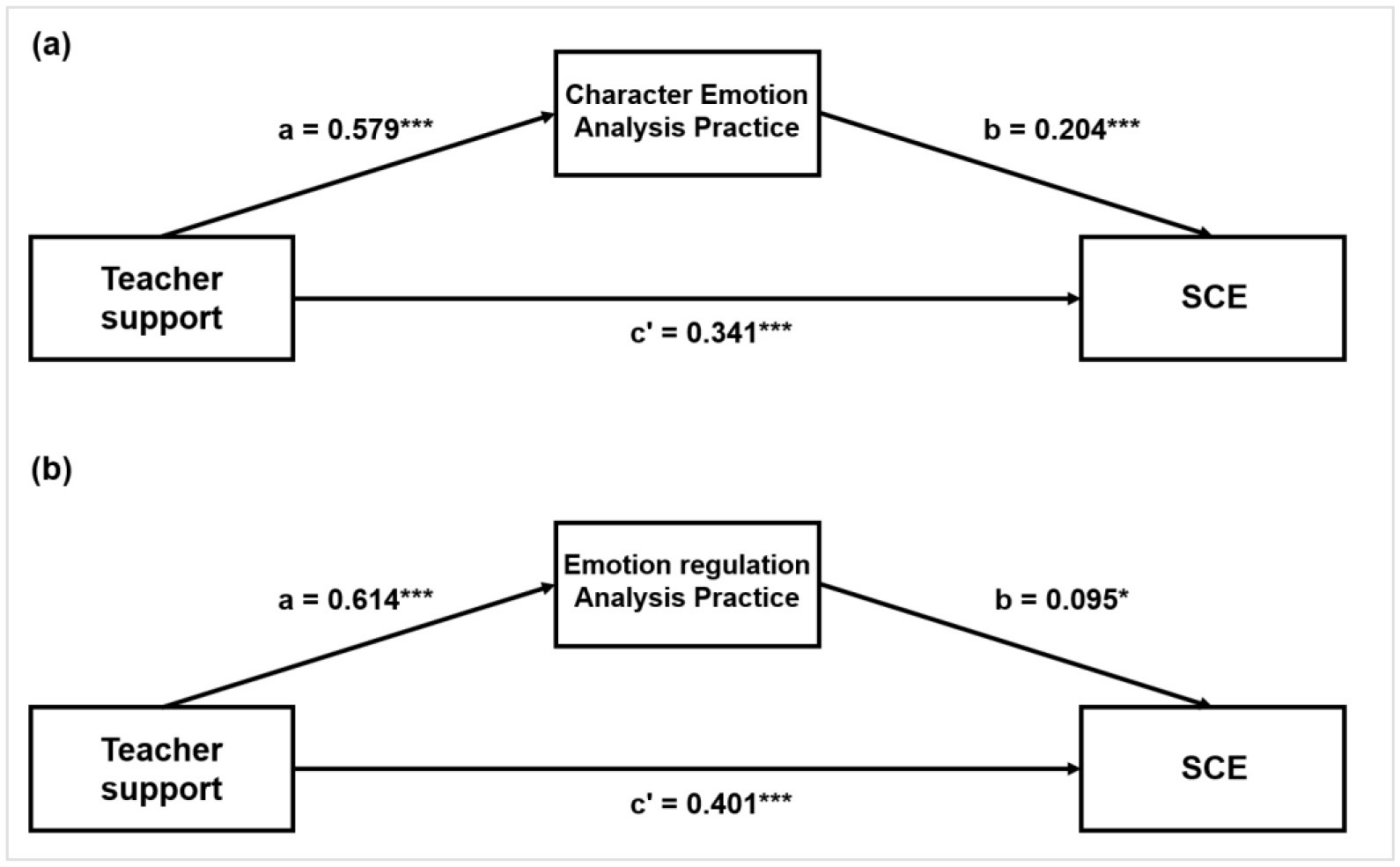
Mediating models of teacher support on social-emotional competencies **(a)** mediation through character emotion analysis practice **(b)** mediation through emotion regulation analysis practice. *p < 0.05, **p < 0.01, ***p < 0.001.

Mediation analyses were conducted to examine whether students' perceptions of instructional practices were statistically associated with the relationship between Teacher Support and overall SEC. Four potential mediators were examined: two formal curricular courses (Moral and Legal Education and Psychological Health Education) and two types of perceived teacher-led reading activities (Character Emotion Analysis and Emotion Regulation Instruction). The results are summarized below and depicted in Figure 2.

For Moral and Legal Education, Teacher Support was significantly associated with both SEC (*c* = 0.443, 95% CI [0.372, 0.513], *p* < 0.001) and the mediator (*a* = 0.517, 95% CI [0.403, 0.635], *p* < 0.001). However, the mediator was not significantly associated with SEC when controlling for Teacher Support (*p* = 0.206), resulting in a nonsignificant indirect effect.

For Psychological Health Education, Teacher Support was strongly associated with the mediator (*a* = 0.782, 95% CI [0.627, 0.934], *p* < 0.001), but the mediator was not significantly associated with SEC (*p* = 0.577), yielding a nonsignificant indirect effect.

In contrast, both perceived reading practices showed significant indirect associations. For Character Emotion Analysis, Teacher Support was significantly associated with the mediator (*a* = 0.579, 95% CI [0.482, 0.676], *p* < 0.001), and the mediator was significantly associated with SEC (*b* = 0.204, 95% CI [0.141, 0.266], *p* < 0.001). The indirect effect was statistically significant (*ab* = 0.118, 95% CI [0.078, 0.162], *p* < 0.001), as was the direct effect (*c*′ = 0.341, 95% CI [0.272, 0.409], *p* < 0.001), indicating partial mediation.

For Emotion Regulation Instruction, a similar pattern emerged. Teacher Support was significantly associated with the mediator (*a* = 0.614, 95% CI [0.517, 0.711], *p* < 0.001), and the mediator was significantly associated with SEC (*b* = 0.095, 95% CI [0.015, 0.173], *p* = 0.022). The indirect effect was statistically significant (*ab* = 0.058, 95% CI [0.009, 0.109], *p* = 0.022), alongside a significant direct effect (*c*′ = 0.401, 95% CI [0.325, 0.479], *p* < 0.001).

These results indicate that students' perceptions of teacher-led reading activities showed significant statistical associations with the Teacher Support–SEC relationship, whereas perceptions of formal curricular courses did not.

## Discussion

The present study examined ecological factors associated with social-emotional competencies among primary school students in rural XiZang, with particular attention to variation across grade levels and student background characteristics. By situating social-emotional development within a culturally distinctive and resource-constrained educational context, the findings extend existing educational psychology research that has been largely concentrated in urban and mainstream settings. At the same time, the study highlights important methodological and interpretive considerations for future research on social-emotional competencies in minority and rural populations.

### Measurement properties and cross-cultural applicability

The social-emotional competencies and Ecological Support Factors scales demonstrated strong psychometric properties in the rural XiZang sample. Both exploratory and confirmatory factor analyses supported the hypothesized six-factor structure of social-emotional competencies and the five-factor structure of ecological support factors. Although widely used social-emotional learning instruments such as the Social Emotional Assets and Resilience Scale and the Social and Emotional Competence Questionnaire have been validated primarily in urban or culturally dominant educational contexts, the present findings suggest that core dimensions of social-emotional competence, including emotional regulation, prosocial values, and self-awareness, can be reliably assessed among students navigating bilingual instruction and culturally distinctive community norms.

This finding is consistent with prior research indicating that fundamental aspects of emotional and social functioning may exhibit cross-cultural commonality, even when their behavioral expressions are shaped by local cultural practices and values (Boyatzis, 2018). At the same time, quantitative self-report measures cannot fully capture the situational and interactional nuances through which social-emotional competencies are enacted in daily life. Future research incorporating qualitative interviews, classroom observations, or culturally grounded behavioral assessments would help clarify how these competencies manifest in the lived experiences of rural XiZang students, thereby strengthening the cultural validity and applied relevance of social-emotional assessment in minority educational contexts.

### Ecological supports and social-emotional competencies

Consistent with a substantial body of educational psychology research emphasizing the importance of teacher-student relationships for social-emotional development (Roorda et al., 2011; Quin, 2017; Zee and Koomen, 2016), Teacher Support emerged as the most robust and consistent ecological correlate of students' social-emotional competencies across multiple dimensions. This pattern aligns closely with ecological systems theory, which emphasizes proximal processes within the microsystem, particularly sustained interactions with significant adults, as central mechanisms of development (Bronfenbrenner, 1979; Neal and Neal, 2013). In the rural XiZang context, where material resources are often limited and cultural norms emphasize respect for teacher authority, supportive teacher behavior may play an especially salient role in shaping students' emotional regulation, interpersonal skills, and self-awareness (see also Dyson et al., 2023).

School Support was also positively associated with several dimensions of social-emotional competencies, consistent with prior findings that supportive institutional environments promote students' sense of belonging, emotional security, and prosocial behavior (Wang and Degol, 2016; Bear et al., 2015). Together, these results underscore the importance of both relational and institutional dimensions of the school ecology for social-emotional development, even in contexts characterized by structural constraints.

Grade stratified analyses revealed meaningful developmental differences in the relative importance of ecological supports. Among fourth and fifth grade students, Teacher Support remained the strongest correlate of social-emotional competencies. In contrast, among sixth grade students, Class Management and Family Support emerged as more prominent correlates, whereas Teacher Support was no longer significantly associated with overall social-emotional competencies. These patterns are consistent with developmental research suggesting that as children approach early adolescence, they experience shifts in social roles, cognitive capacities, and sources of influence, with increasing attention to classroom structure, autonomy, and family expectations (Eccles and Roeser, 2011; Wubbels and Brekelmans, 2012). The finding that sixth grade students demonstrated higher levels of emotional understanding relative to younger students further aligns with evidence that cognitive maturation supports more differentiated emotional reasoning and perspective-taking during this developmental period (Rivers et al., 2013).

From an applied perspective, these findings suggest that effective support strategies for social-emotional competencies may need to be developmentally differentiated. While relational warmth and emotional responsiveness from teachers may be especially critical in earlier grades, structured classroom management and family-based supports may assume greater importance as students' progress through primary school.

The observed associations between family economic status and selected social-emotional competencies, particularly prosocial values and emotional understanding, are consistent with prior research linking socioeconomic resources to social-emotional development through pathways such as environmental stability, access to enriching experiences, and reduced stress exposure (Bradley and Corwyn, 2002; Hopson and Lee, 2011). These findings highlight the importance of situating social-emotional development within broader socioeconomic contexts, especially in resource-constrained educational settings.

### Reading experience and social-emotional competencies

Students who reported prior exposure to emotion-themed picture books demonstrated higher levels of social-emotional competencies across most dimensions. This pattern is consistent with theoretical accounts emphasizing the role of narrative engagement in fostering empathy, emotional understanding, and perspective-taking (Mar et al., 2011; Kidd and Castano, 2013). In the rural XiZang context, where formal social-emotional learning curricula may be limited or unevenly implemented, emotionally rich reading materials may represent an accessible avenue for supporting social-emotional learning within everyday instructional practices.

However, these findings must be interpreted with appropriate caution. Because reading experience was assessed through self-report rather than experimental manipulation, the observed associations may reflect selection effects, shared underlying factors, or reverse directionality. Future research employing longitudinal designs or randomized controlled interventions would be necessary to establish whether exposure to emotion-themed reading materials directly contributes to the development of social-emotional competencies.

### Student profiles and heterogeneity

The cluster analysis identified three distinct student profiles characterized by different configurations of social-emotional competencies and ecological support factors, namely a High Support High Competency group, a Moderate Support Moderate Competency group, and a Low Support Low Competency group. Membership in these profiles was significantly associated with family economic status, parental marital status, and reading experience. These findings underscore the substantial heterogeneity that exists within the rural XiZang student population and suggest that children's social-emotional development is shaped by constellations of ecological conditions rather than by isolated factors (Scales, 2011).

From a theoretical perspective, the emergence of these profiles is consistent with ecological and developmental frameworks that emphasize the cumulative and interactive effects of supports across multiple contexts. Students experiencing consistently high levels of support across school and family environments tended to demonstrate stronger social-emotional competencies, whereas those exposed to multiple forms of disadvantage were more likely to fall into the low support low competency profile. From a practical standpoint, the identification of a subgroup characterized by both limited ecological support and lower social-emotional competencies highlights the potential value of targeted and differentiated support strategies for students who may be at elevated risk. At the same time, the present findings are descriptive in nature, and the effectiveness of profile-specific interventions would need to be evaluated through carefully designed longitudinal or experimental research.

### Perceived instructional practices and teacher support

The mediation analyses examined whether students' perceptions of different instructional practices were statistically associated with the relationship between Teacher Support and overall social-emotional competencies. Students' perceptions of teacher-guided reading activities, specifically Character Emotion Analysis and Emotion Regulation Instruction, demonstrated significant indirect associations. In contrast, perceptions of formal curricular courses, including Moral and Legal Education and Psychological Health Education, were not significantly associated with this relationship. This pattern suggests that, from students' perspectives, emotionally focused reading activities embedded within everyday classroom interactions may represent a more salient pathway through which supportive teacher relationships are linked to social-emotional competencies than more formalized curricular instruction.

Several interpretive cautions should be noted. As discussed in the Limitations section, the cross-sectional design precludes causal inference, and the assumed temporal ordering cannot be empirically verified (Maxwell and Cole, 2007). Additionally, the instructional practice variables reflected students' subjective perceptions rather than objective indicators of instructional quality or fidelity, raising the possibility of shared method variance. Finally, the absence of significant indirect associations for formal curricular courses should not be interpreted as evidence that such courses are ineffective, as their content, pedagogical quality, and implementation consistency were not directly assessed.

Despite these limitations, the overall pattern of findings is theoretically informative. It aligns with perspectives emphasizing that interactive, experiential, and contextually meaningful pedagogical practices may be more closely connected to social-emotional development than didactic or highly formalized instruction (Durlak et al., 2011). In resource-constrained educational settings, where the implementation of comprehensive social-emotional learning curricula may be challenging, teacher-guided reading activities that engage students in emotional reflection and discussion may represent a feasible and promising approach, consistent with findings from other resource-constrained educational contexts (Lee, 2023). These findings point to the need for future intervention studies that systematically examine whether such practices can be effectively leveraged to support students' social-emotional development across diverse cultural and educational contexts.

### Implications for practice

While acknowledging the correlational nature of the findings, several tentative implications for educational practice may be considered. The consistent associations observed between Teacher Support and Social-emotional competencies across multiple dimensions suggest that efforts to strengthen teacher-student relationships may be relevant for supporting students' social-emotional development. Professional development initiatives that emphasize responsive teaching, emotional attunement, and positive classroom interactions may be particularly beneficial, especially in educational contexts where teachers serve as primary sources of guidance and emotional support for students (Oberle et al., 2020).

The observed associations between reading experience and social-emotional competencies, together with the mediation findings related to teacher-guided reading activities, suggest that the integration of emotion-themed reading materials and structured discussion into regular classroom instruction warrants further consideration. Such approaches typically require limited specialized resources and can be adapted to local cultural narratives and linguistic practices, potentially offering practical advantages in resource-constrained educational settings. At the same time, the effectiveness of these approaches cannot be assumed based on correlational evidence alone and would need to be established through rigorously designed intervention studies before specific programmatic recommendations are made.

The developmental patterns identified across grade levels further suggest that social-emotional support strategies may need to be differentiated according to students' developmental stages. For older primary school students, greater attention to classroom management practices and family engagement may be particularly relevant. In addition, the cluster analysis findings point to the potential value of identifying students who experience multiple forms of ecological disadvantage and directing targeted supports toward those students who may be at elevated risk for lower social-emotional competencies.

### Limitations and future directions

Several limitations of the present study should be acknowledged. First, the cross-sectional design precludes causal inference. All associations reported in this study, including those derived from regression and mediation analyses, reflect concurrent relationships and do not establish that ecological supports, instructional practices, or reading experiences cause changes in social-emotional competencies. Longitudinal research tracking students over time, as well as experimental studies manipulating specific supports or pedagogical practices, would be necessary to draw causal conclusions.

Second, reading experience was assessed through students' retrospective self-reports of whether they had previously read emotion-themed picture books. Students were not randomly assigned to reading conditions, and no information was available regarding the frequency, duration, recency, or instructional context of these reading experiences. As a result, the observed association between reading experience and social-emotional competencies may reflect selection effects, unmeasured confounding variables, or reverse directionality rather than a causal influence of reading on social-emotional development.

Third, the instructional practice variables captured students' subjective perceptions rather than objective indicators of instructional content or implementation quality. No data were collected regarding the actual structure, fidelity, or pedagogical features of teacher-guided reading activities or formal curricular courses. Future research incorporating classroom observation, teacher reports, or standardized instructional protocols would provide a more robust basis for evaluating the role of specific instructional practices.

Fourth, all data were obtained through student self-report, raising the possibility of common method variance and social desirability bias. The inclusion of multiple informants, such as teachers and parents, as well as multiple methods, including behavioral observation or performance-based assessments, would strengthen future investigations.

Fifth, the sample was drawn exclusively from rural primary schools in Lhasa, XiZang. As such, the generalizability of the findings to other regions, cultural contexts, or educational systems may be limited. Replication studies in other minority and rural communities, as well as comparative research across diverse cultural settings, would help clarify the boundaries within which the present findings apply.

Despite these limitations, the study provides a systematic examination of ecological factors associated with social-emotional competencies in an educational context that has received limited empirical attention. The findings offer a foundation for generating hypotheses to be tested in future longitudinal and intervention research and provide preliminary insights that may inform the development of culturally responsive approaches to supporting social-emotional development in rural and minority educational settings.

## Conclusion

The present study examined associations among ecological support factors, reading experiences, perceived instructional practices, and social-emotional competencies among primary school students in rural XiZang. Teacher Support and School Support demonstrated consistent positive associations with social-emotional competencies, with evidence of developmental variation in the salience of different ecological factors across grade levels. Students who reported prior exposure to emotion-themed picture books exhibited higher levels of social-emotional competencies, and students' perceptions of teacher-guided reading activities were statistically associated with the relationship between Teacher Support and social-emotional competencies in mediation analyses.

By extending educational psychology research to a culturally distinctive and underrepresented educational context, this study contributes to a more inclusive understanding of the ecological conditions associated with children's social-emotional development. At the same time, the findings highlight the need for future longitudinal and experimental research to clarify the causal nature of these associations and to evaluate the effectiveness of specific pedagogical and ecological support strategies. Such work will be essential for informing the design of developmentally appropriate and culturally responsive interventions aimed at promoting social-emotional competencies among rural and minority student populations.

## Data Availability

The raw data supporting the conclusions of this article will be made available by the authors, without undue reservation.
